# Bioinformatics Approach to Identify the Pathogenetic Link of Gut Microbiota-Derived Short-Chain Fatty Acids and Ischemic Stroke

**DOI:** 10.1007/s12035-024-04176-7

**Published:** 2024-04-22

**Authors:** Liang Ding, Jianing Wang, Sha Qiu, Zhizhen Ren, Yuantao Li, Pengpeng An

**Affiliations:** 1 Department of Traditional Chinese Medicine, Qingdao Third People’s Hospital, Qingdao City, Shandong Province China; 2 Neurology Department, Qingdao Hiser Hospital Affiliated of Qingdao University (Qingdao Traditional Chinese Medicine Hospital), Qingdao City, Shandong Province China; 3 Department of Traditional Chinese Medicine, Qingdao Central Hospital, University of Health and Rehabilitation Sciences (Qingdao Central Hospital), Qingdao City, Shandong Province China; 4 Department of Traditional Chinese Medicine, Community Health Service Center of Shi’nan District in Qingdao, Qingdao City, Shandong Province China; 5 Acupuncture and Moxibustion Department, Qingdao Third People’s Hospital, Qingdao City, Shandong Province China; 6 Emergency Internal Medicine Department, Qingdao Hiser Hospital Affiliated of Qingdao University (Qingdao Traditional Chinese Medicine Hospital), Qingdao City, Shandong Province China

**Keywords:** Ischemic stroke, Short-chain fatty acids, Gut-brain axis, Bioinformatics analysis, Immune response

## Abstract

Stroke is a life-threatening condition that impairs the arteries and causes neurological impairment. The incidence of stroke is increasing year by year with the arrival of the aging population. Thus, there is an urgent need for early stroke diagnosis. Short-chain fatty acids (SCFAs) can modulate the central nervous system and directly and indirectly impact behavioral and cognitive functions. This study aimed to investigate the connection between SCFA metabolism and stroke development via bioinformatic analysis. Initially, the Gene Set Enrichment Analysis (GSEA) and immune cell infiltration analysis were performed based on RNA data from stroke patients to comprehend the mechanisms governing stroke pathogenesis. The functional analysis, including Gene Ontology (GO), Kyoto Encyclopedia of Genes and Genomes (KEGG), and Protein-Protein Interaction (PPI), was performed based on the Differentially Expressed Gene (DEG) selected by the limma package. 1220 SCFA metabolism-related genes screened from Genecards databases were intersected with 242 genes in main modules determined by Weighted Gene Co-Expression Network Analysis (WGCNA), and the final 10 SCFA key genes were obtained. GO analysis revealed that these genes were involved in immune response processes. Through lasso regression analyses, we established a stroke early diagnosis model and selected 6 genes with diagnostic value. The genes were validated by the area under curve (AUC) values and had a relatively good diagnostic performance. Finally, 4 potential therapeutic drugs targeting these genes were predicted using the Drug Signatures Database (DSigDB) via Enrichr. In conclusion, this paper analyzes the involvement of SCFAs in the complex gut-brain axis mechanism, which contributes to developing new targets for treating central nervous system diseases and provides new ideas for early ischemic stroke diagnosis.

## Introduction

Stroke is a general term for a group of sudden localized disorders of cerebral blood circulation resulting in neurological dysfunction [1]. Approximately 80% of all strokes are ischemic and usually caused by thrombosis or embolism. Hemorrhagic strokes, caused by blood vessel ruptures (e.g., subarachnoid hemorrhage and cerebral hemorrhage), account for about 20% of strokes. Transient stroke symptoms (usually lasting 1 h) without evidence of acute cerebral infarction (based on diffusion-weighted MRI) are referred to as transient ischemic attack. With the aging of the population, the incidence of ischemic stroke is increasing annually [2, 3]. In the United States, stroke ranks as the fifth leading cause of death and the most widespread disabling neurologic condition in adults. It is of great concern in the medical and scientific fields as it poses a severe threat to human health with its continuously increasing incidence, disability, mortality, and recurrence rates [4, 5].

After a stroke, the brain undergoes neuroinflammation, oxidative stress, and excitotoxicity. Of these, neuroinflammation is at the core of secondary brain injury, which can persist for days or even weeks and have a significant impact on stroke prognosis [6]. In addition to intracerebral lesions, a wide range of extracerebral lesions occur, including considerable gut flora and systemic immune dysregulation, which can counteract the central nervous system and exacerbate brain damage. Gastrointestinal dysfunction is one of the most common complications in stroke patients. Although clinical symptoms resulting from gastrointestinal dysfunction are not intense or prominent [7–9], such dysfunction does affect the patients’ nutrient intake and may trigger intestinal barrier dysfunction, which in turn triggers systemic changes such as bacterial translocation and ultimately affects the overall prognosis of patients [10–12]. Clinical studies have found that depression, anxiety, and other psychiatric symptoms often occur in conjunction with intestinal symptoms. The occurrence and prognosis of these conditions can be regulated by the principle of the brain-gut axis. Suggest that the intestinal flora and its metabolites are capable of exchanging information bidirectionally with both the central nervous system (CNS) and enteric nervous system through direct or indirect pathways, leading to effects on multiple systems such as neurology, endocrinology, and immunity [13–15]. Therefore, intervening in the metabolism of intestinal flora can mediate central nervous function and the prognosis of stroke.

**Fig. 1:**
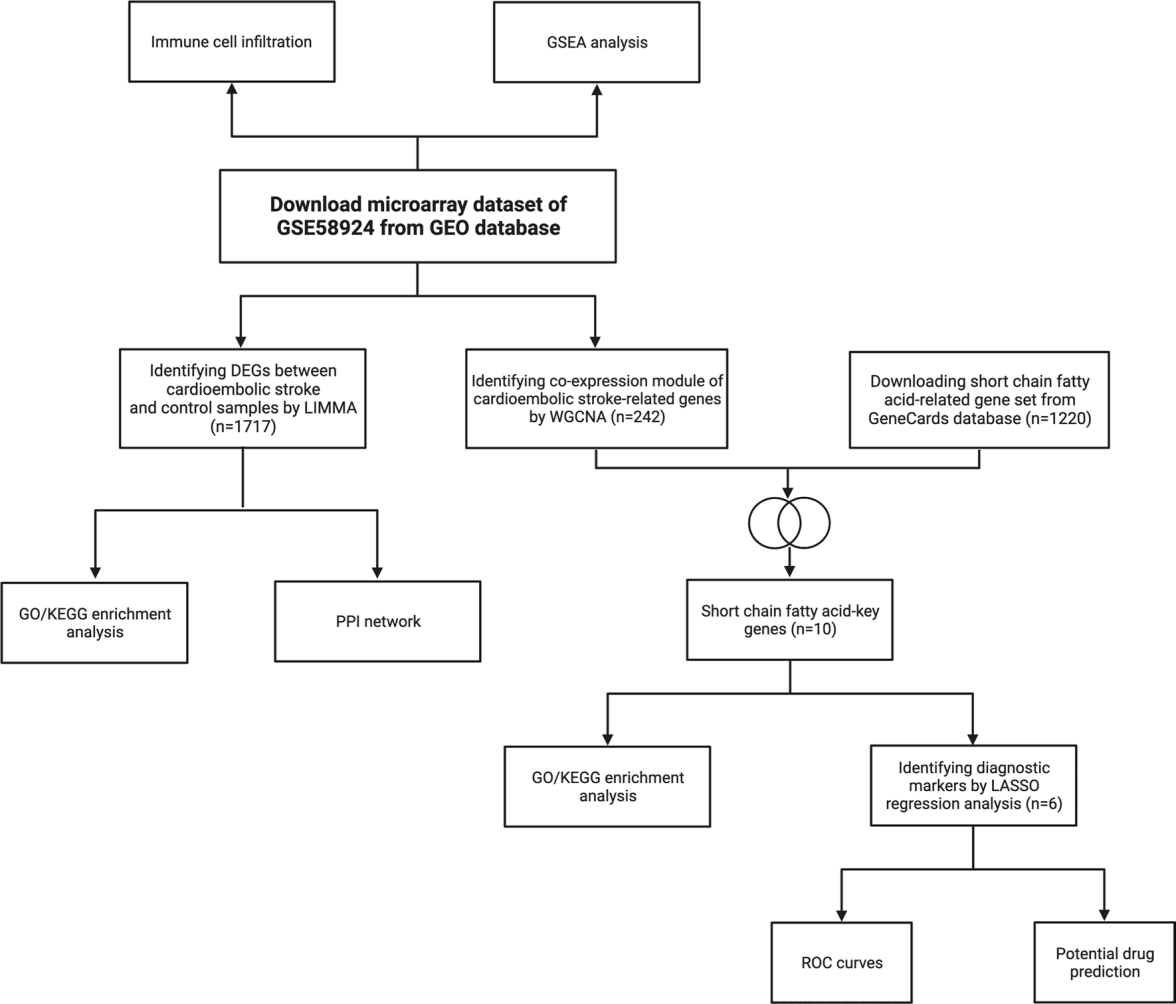
Study flow chart of present work

The intestinal flora produces metabolites such as lipopolysaccharides, peptidoglycans, trimethylamine, secondary bile acids, and short-chain fatty acids by acting on undigested food and endogenous mucus secreted by intestinal epithelial cells. These metabolites can have both harmful and beneficial effects on the human body. They are absorbed through the intestine and can affect the metabolic health of the host. Short-chain fatty acids (SCFAs) are the most commonly studied small-molecule metabolites produced by intestinal microbial fermentation of dietary fiber. They include formic acid, acetic acid, propionic acid, butyric acid, valeric acid, hexanoic acid, and their isomers. Acetic acid, propionate, and butyrate are the most abundant SCFAs in the human body. Due to the abundant expression of SCFA transporter proteins on endothelial cells, SCFAs are able to penetrate the blood-brain barrier, access the brain, and affect inflammation in the CNS through the enteric nervous system [16–18]. Furthermore, they possess the potential to directly or indirectly regulate neuroimmune, improve neuronal function, and potentially alleviate or treat neurodegenerative diseases and cerebral diseases. Contribute to the communication of the gut-brain axis through multiple potential pathways, including endocrine, immunomodulatory, vagal, and humoral pathways. Therefore, restoring a healthy microbial ecology in the gut may be a critical therapeutic goal for effectively managing and treating ischemic stroke, and developing gut-brain axis mechanisms could potentially introduce novel therapeutic targets to neurodegenerative diseases. Nevertheless, the current research on gut-brain axis mechanisms is insufficient to draw definitive conclusions. This article will address the role of SCFAs in the process of stroke. First, we obtained the stroke gene expression profiles from the Gene Expression Omnibus (GEO) database. The pathogenesis and immune cell composition related to the stroke process were performed using Gene Set Enrichment Analysis (GSEA) and immune cell infiltration analysis. The genes associated with the progression of stroke were screened by limma and further evaluated by Gene Ontology (GO), Kyoto Encyclopedia of Genes and Genomes (KEGG), and protein–protein interaction (PPI) network analysis. The hub genes screened by weighted gene co-expression network (WGCNA) analysis were intersected with SCFA-related genes, and 6 diagnostic marker genes were identified by machine learning algorithms and verified by receiver operating characteristic (ROC) analysis. The potential therapeutic drugs targeting these genes were also predicted using the Drug Signatures Database (DSigDB) via Enrichr. The flow chart of this study is presented in Fig. 1. This study provides a basis for new scientific evidence for the association between intestinal flora and stroke and offers new targets for treating central nervous system diseases.

## Method

### Data Collection

First, we identified SCFA metabolism-related genes from the GeneCards Database (https://www.genecards.org/) by using “acetic acid, butyrate, propionic acid, and short-chain fatty acid” as search terms. Subsequently, through the GEO database (https://www.ncbi.nlm.nih.gov/geo/), we download the transcriptional data profiles for 69 cardioembolic stroke and 23 vascular risk factor controls without symptomatic vascular diseases (GEO ID: GSE58924).

### Immune Cell Infiltration

The immune infiltration in stroke was calculated by examining the signature of 22 kinds of immune cell expression spectrum matrix using the CIBERSORTx website (https://cibersortx.stanford.edu/). The relative percent, correlation index, and root-mean-squared prediction error (RMSE) of 22 kinds of immune cell infiltration in stroke patients were plotted.

### GSEA Analysis

GSEA analysis was performed to identify gene sets in cardioembolic stroke. The gene sets of “c2.cp.all.v2022.Hs.symbols.gmt” was used from the MSigDB database (https://www.gsea-msigdb.org/gsea/msigdb/index.jsp). All analyses and visualizations are carried out in R 4.2.1. R packages involved: cluster Profiler package (for enrichment analysis) and msigdbr package (reference gene set source).

### Identification of Differentially Expressed Cardioembolic Stroke-Related Genes

We set |Log_2_fold change (FC)| > 0.585 and adj. *p* < 0.05 as a threshold to screen out differentially expressed cardioembolic stroke-related genes. The limma package in R was used to analyze mRNA expression in cardioembolic stroke and healthy samples. The differentially expressed genes (DEGs) were visualized in volcano maps and heat maps.

### WGCNA Analysis and Module Gene Selection

Throughout the entirety of the process of constructing a scale-free co-expression network, the WGCNA package was utilized. First, we calculated the median absolute deviation (MAD) of each gene by using a gene expression profile and excluded the top 50% of the smallest genes. The outlier genes and samples were removed by the goodSamplesGenes method of the R software package WGCNA. Then, Pearson correlation matrices and the average linkage method were performed for all pairwise genes. Then, a weighted adjacency matrix was constructed using a power function *A*_mn=/*C*_mn| ^ *β*. After choosing the power of 7, the adjacency was transformed into a topological overlap matrix (TOM), and the corresponding dissimilarity (1-TOM) was calculated. To classify genes with similar expressions into gene modules, average linkage hierarchical clustering was conducted according to the TOM dissimilarity. To further analyze the module, the sensitivity was set to 3 to calculate the dissimilarity of module eigengenes, choose a cut line for the module dendrogram, and merge some modules [19].

### Function Enrichment Analysis

Functional enrichment analysis was performed to explore the potential biological implications. GO enrichment and KEGG pathway analysis were performed using the “clusterProfiler” and “org.Hs.eg.db” R packages. The BH method used adj. *p* as the threshold in GO enrichment and KEGG pathway analysis. The biological process (BP), molecular function (MF), and cellular component (CC) categories of DEGs were performed using a David online tool (https://david.ncifcrf.gov/). KEGG pathway enrichment analysis was performed using the KOBAS 3.0 online analysis database (http://kobas.cbi.pku.edu.cn/) [20]. The results of functional enrichment analysis were considered significantly enriched if the adj. *p* < 0.05. In addition, the top 5 GO terms and top 10 KEGG pathways are shown visually in the bubble chart. To excavate interactions among the DEGs, the PPI network was constructed using the Search Tool for the Retrieval of Interacting Genes Database (STRING) (https://cn.string-db.org/), with the high confidence values (confidence scores > 0.700). Subsequently, the PPI network was visualized by Cytoscape software, and an MCODE plug-in was used to screen the hub genes [21–23].

### Identification of the SCFA-Related Hub Genes by LASSO Logistic Regression

The Least Absolute Shrinkage Selection Operator (LASSO) analysis was adopted to further identify the SCFA-related hub genes of early stroke diagnosis [24]. Statistical analysis and visualization were performed in R 4.2.1. glmnet package was used to analyze the cleaned data to obtain variable lambda value, likelihood value classification error rate, etc. The minimum lambda was defined as the optimal value.

### Identification of the Diagnostic Values of SCFA-Related Hub Genes by ROC Analysis

The ROC curve was utilized to evaluate the diagnostic performance of SCFA-related hub genes. The accuracy level of the area under the curve (AUC) can be classified as low (0.5–0.7), moderate (0.7–0.9), and high (above 0.9), with an AUC closer to 1, indicating a superior diagnostic efficacy. pROC [1.18.0] is used for ROC analysis and ROC verification. Statistical analysis and visualization were performed in R 4.2.1.

### Potential Drug Prediction

Potential therapeutic drugs based on the potential diagnostic markers of stroke were predicted using the DSigDB via Enrichr, with an adj. *p* < 0.05 being selected as criteria.

### Statistical Analysis

All statistical analyses were conducted using R software. Analysis of variance (ANOVA) or *t*-tests was used to investigate significant differences between indicated groups. *p* < 0.05 was considered statistically significant.

## Results

### Immune Cell Infiltration Analysis in Cardioembolic Stroke

**Fig. 2:**
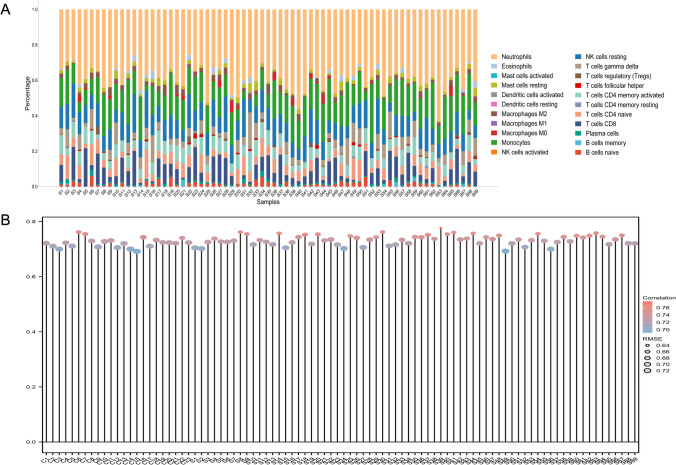
Immune cell infiltration. **A** The relative percentage of 22 immune cells in the cardioembolic stroke was calculated by the CIBERSORT algorithm. **B** The correlation and RMSE of 22 immune cell infiltration in stroke and healthy control was visualized in lollipop plots. C, control group; S, stroke group

In the early stage of stroke, necrotic cells and damaged neurons release damage-associated molecular patterns (DAMPs) to induce increased glial activation, peripheral immune response, and secretion of inflammatory mediators. This process accelerates blood-brain barrier disruption, exacerbates cerebral oedema, and causes microcirculatory disturbances. The immune response has been shown to participate in this pathophysiological progression of stroke [25]. To understand the immune cell composition in cardioembolic stroke. Twenty-two kinds of immune cell infiltration analysis on GEO transcriptome sequencing data were evaluated. According to the results, the relative percent of immune cells revealed that in cardioembolic stroke patients, neutrophils, monocytes, and NK resting cells with higher percentages, NK activated cells, activated mast cells, and macrophage M1 with lower percentages (Fig. 2A), correlation analysis showed that compared to the control group, the cardioembolic stroke patients have higher correlation and lower RMSE value (the smaller the RMSE, the better the prediction). The correlation and RMSE of 22 kinds of immune cell infiltration in cardioembolic stroke were visualized in lollipop plots (Fig. 2B). Research has demonstrated that after a stroke, brain tissue triggers an inflammatory response. This leads to the activation of neutrophils, which migrate to the ischemic area and initiate innate immunity. Unfortunately, this defense overload can also cause damage to the brain tissue and blood vessels. Our analysis has reached the same conclusion as the elevated percentage of neutrophils [26].

### GSEA Analysis in Cardioembolic Stroke

**Fig. 3:**
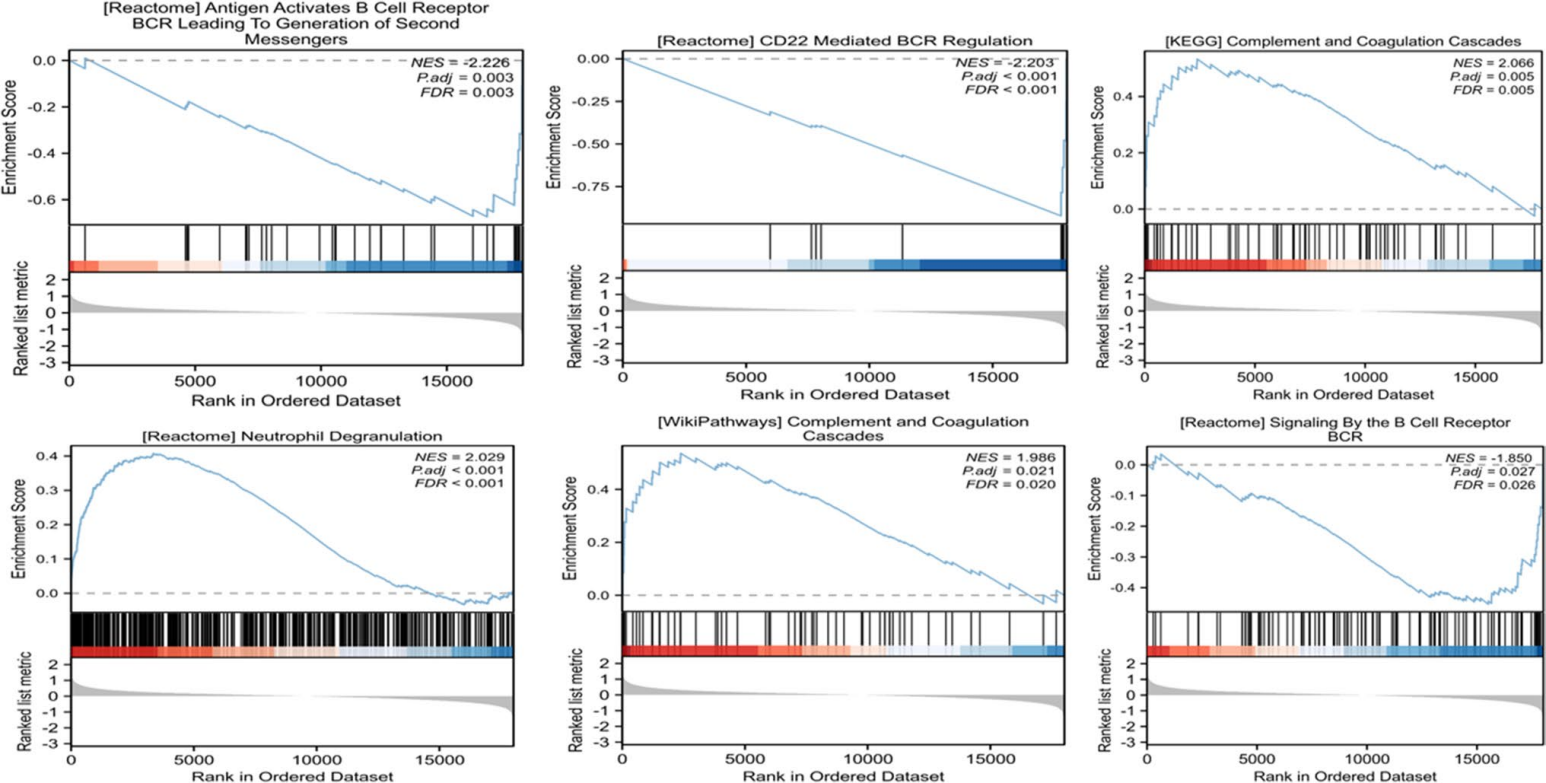
GSEA analysis in cardioembolic stroke

To understand the basic mechanisms of cardioembolic stroke pathogenesis, the enriched gene set in stroke was determined by GSEA using 2 criteria: the adj. *p* < 0.05 and FDR < 0.25. Seven enriched gene sets were obtained, including neutrophil degranulation (NES = 2.029), cd22 mediated B cell receptor regulation (NES = -2.203), antigen activates B cell receptor leading to generation of second messengers (NES = − 2.226), extrafollicular B cell activation by sarscov2 (NES = − 2.074), complement and coagulation cascades (NES = 2.066), complement and coagulation cascades (NES = 1.986), and signaling by the B cell receptor (NES = − 1.85) (Fig. 3). The NES values correspond to the gene set enrichment. A positive value indicates that the gene set is enriched in the high-expression group, and vice versa. GSEA analysis showed that stroke pathogenesis is mainly associated with neutrophil and B cell regulation. This is consistent with previously reported neutrophil extracellular traps (NETs) that regulate ischemic stroke brain injury. The formation of NETs induced by DAMPs in the inflammatory microenvironment via pattern recognition receptor-related signaling cascades increases the risk of thrombosis, which is a crucial pathogenic cause of ischemic stroke [27].

### Identifying the Stroke-Related DEGs in Cardioembolic Stroke

**Fig. 4:**
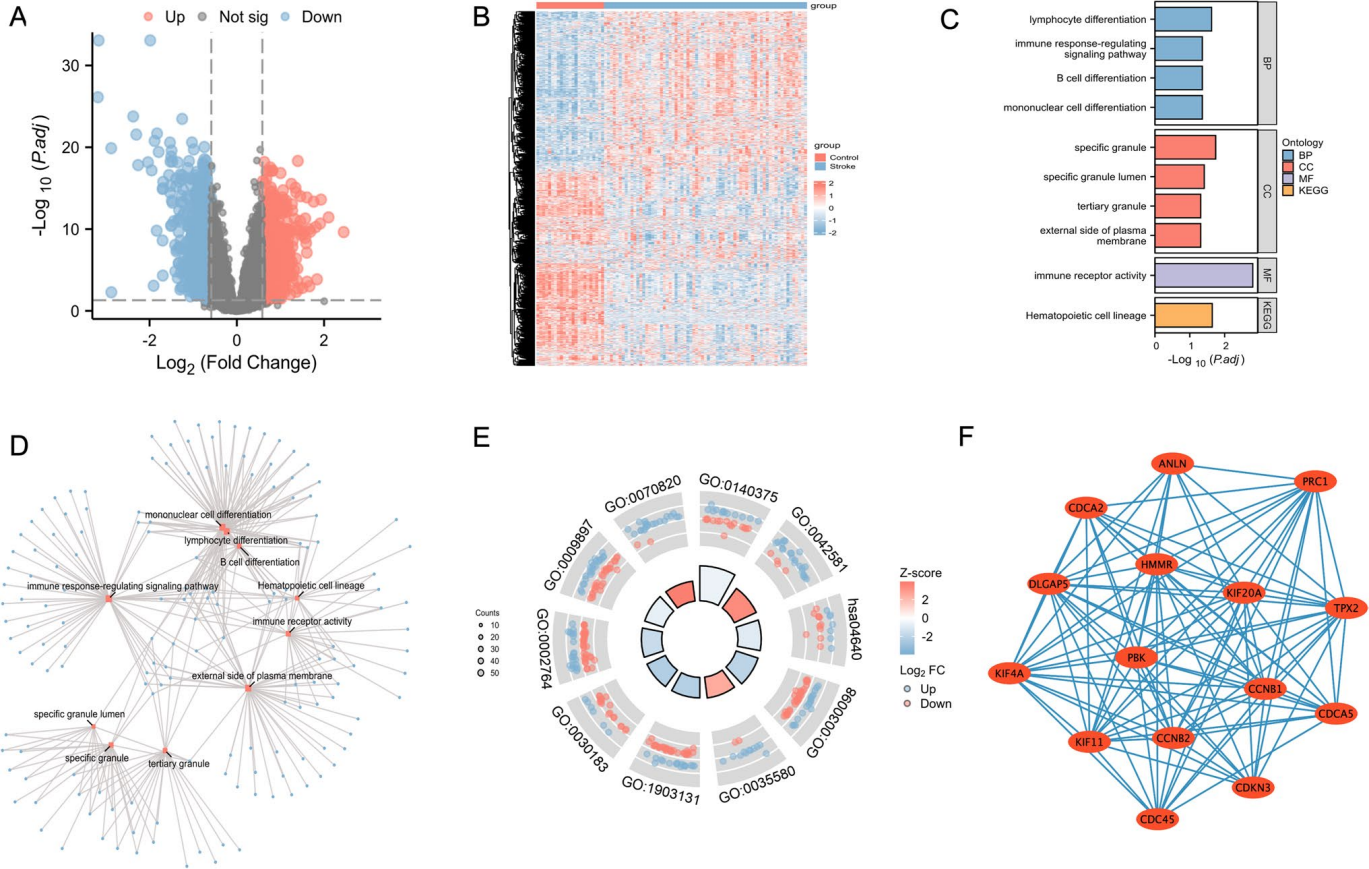
DEGs analysis and functional analyses. **A**, **B** Volcano plot and heatmap showing the DEGs in the cardioembolic stroke of patients. Red and blue represent upregulated and downregulated gene expression. **C**, **D** The bar and network diagram show the GO and KEGG enrichment analysis of DEGs. **E** GO and KEGG enrichment combined with Log_2_FC values of DEGs was demonstrated in a circle diagram. **F** PPI analysis of DEGs

To identify the stroke-related DEGs in cardioembolic stroke, first, the GSE58294 datasets containing 23 healthy samples and 69 cardioembolic stroke samples were used to determine the DEGs with adj. *p* < 0.05, |Log_2_ fold change (FC)| > 0.585 as a cutoff criterion, 1717 DEGs were identified. The corresponding volcano plots and heatmaps were generated to visualize the results (Fig. 4A, B).

GO and KEGG pathway enrichment analysis was performed to further understand the potential biological functions of stroke-related DEGs. The analysis showed that the stroke-related DEGs involved 11 biological processes, including lymphocyte differentiation, mononuclear cell differentiation, B cell differentiation, and immune response-regulating signaling pathway, 4 cellular components, and immune receptor activity molecular function, and mainly involved in the hematopoietic cell lineage pathway. The 4 terms with the largest significant difference were selected and plotted (Fig. 4C, D, Table 1). GO/KEGG functional enrichment combined with Log2FC values is demonstrated in Fig. 4E and Table 1. Based on the provided log2FC, the *z*score was used to determine whether the corresponding term is positively or negatively regulated. The larger the absolute value of the *z*score, the higher the degree of regulation to be. According to the results, the specific granule, specific granule, lumen, and tertiary granule were negatively regulated in the cardioembolic stroke patients, while other enriched pathways were positively regulated.

**Table 1:** GO and KEGG pathway enrichment terms for stroke-related DEGs

Ontology	ID	Description	FDR	Adj. *p*	Gene count	*z*score
BP	GO:0030098	Lymphocyte differentiation	0.023	0.024	48	− 2.021
BP	GO:1903131	Mononuclear cell differentiation	0.042	0.044	50	− 2.263
BP	GO:0030183	B cell differentiation	0.042	0.044	23	− 2.294
BP	GO:0002764	Immune response-regulating signaling pathway	0.042	0.044	54	− 1.633
CC	GO:0042581	Specific granule	0.018	0.018	25	3.4
CC	GO:0035580	Specific granule lumen	0.037	0.038	13	2.496
CC	GO:0009897	External side of plasma membrane	0.047	0.049	49	− 0.714
CC	GO:0070820	Tertiary granule	0.047	0.049	23	3.962
MF	GO:0140375	Immune receptor activity	0.002	0.002	27	− 0.577
KEGG	hsa04640	Hematopoietic cell lineage	0.022	0.023	18	− 0.943

To further reveal the potential relationships between stroke-related DEGs, the PPI network was screened by STRING, including 596 nodes and 1241 edges. Fifteen hub genes, such as KIF20A, CDKN3, TPX2, HMMR, CDCA2, KIF11, CDCA5, CCNB2, PRC1, PBK, DLGAP5, ANLN, CCNB1, KIF4A, and CDC45, were selected and visualized through the MCODE plug-in, and the genes were ranked by node numbers in Fig. 4F.

### Identifying the Co-expression Modules in Stroke by WGCNA

**Fig. 5:**
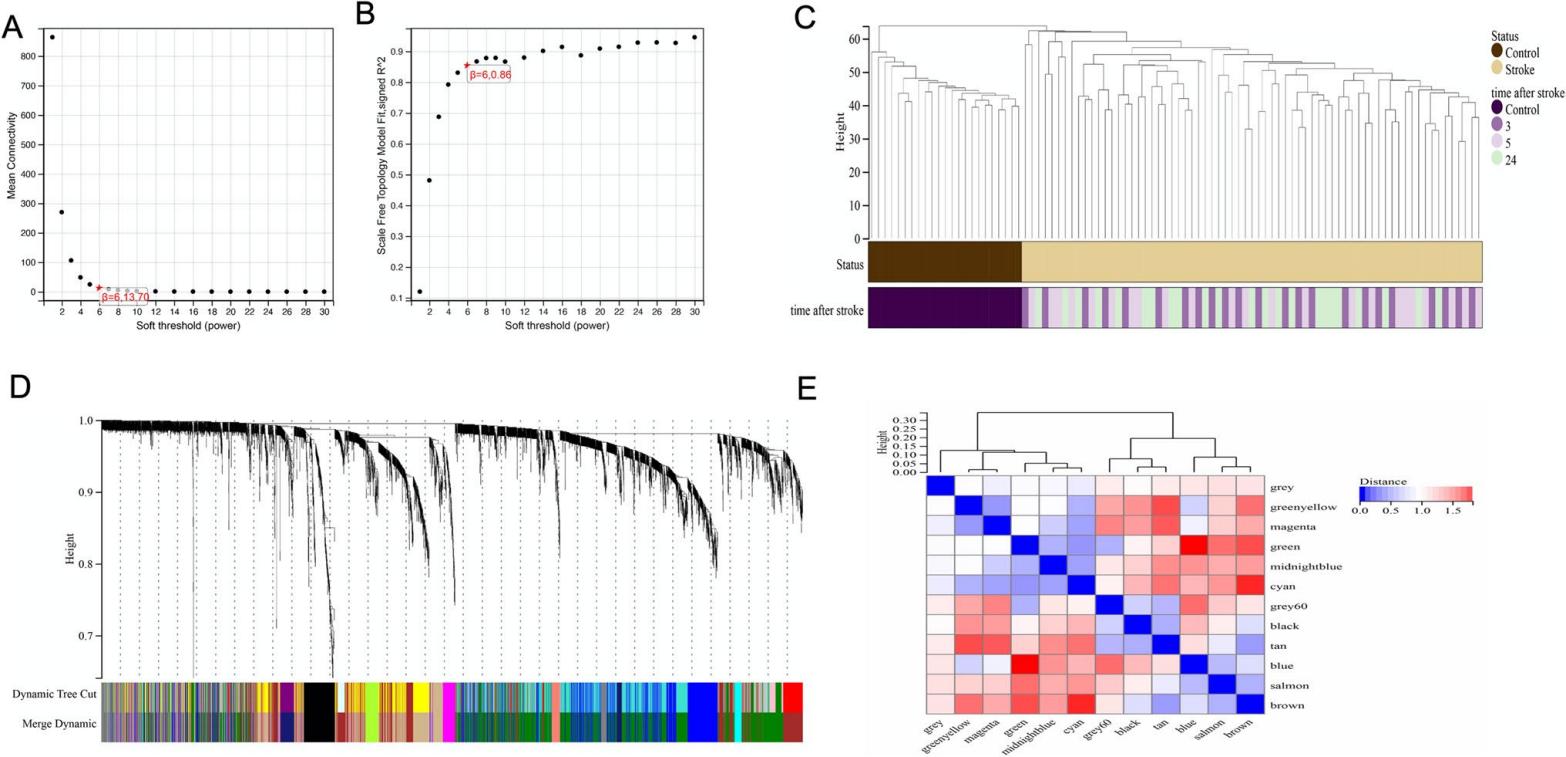
WGCNA. **A**, **B** The corresponding mean connectivity and scale-free topological model fit values at different soft threshold powers. **C** Clustering dendrogram of the stroke and control samples. **D** Cluster dendrogram of genes co-expression modules represented by different colors under the gene tree. **E** Heatmap of the eigengene network representing the relationships between different modules and clinical traits. Red represents a positive correlation, and blue represents a negative correlation

**Fig. 6:**
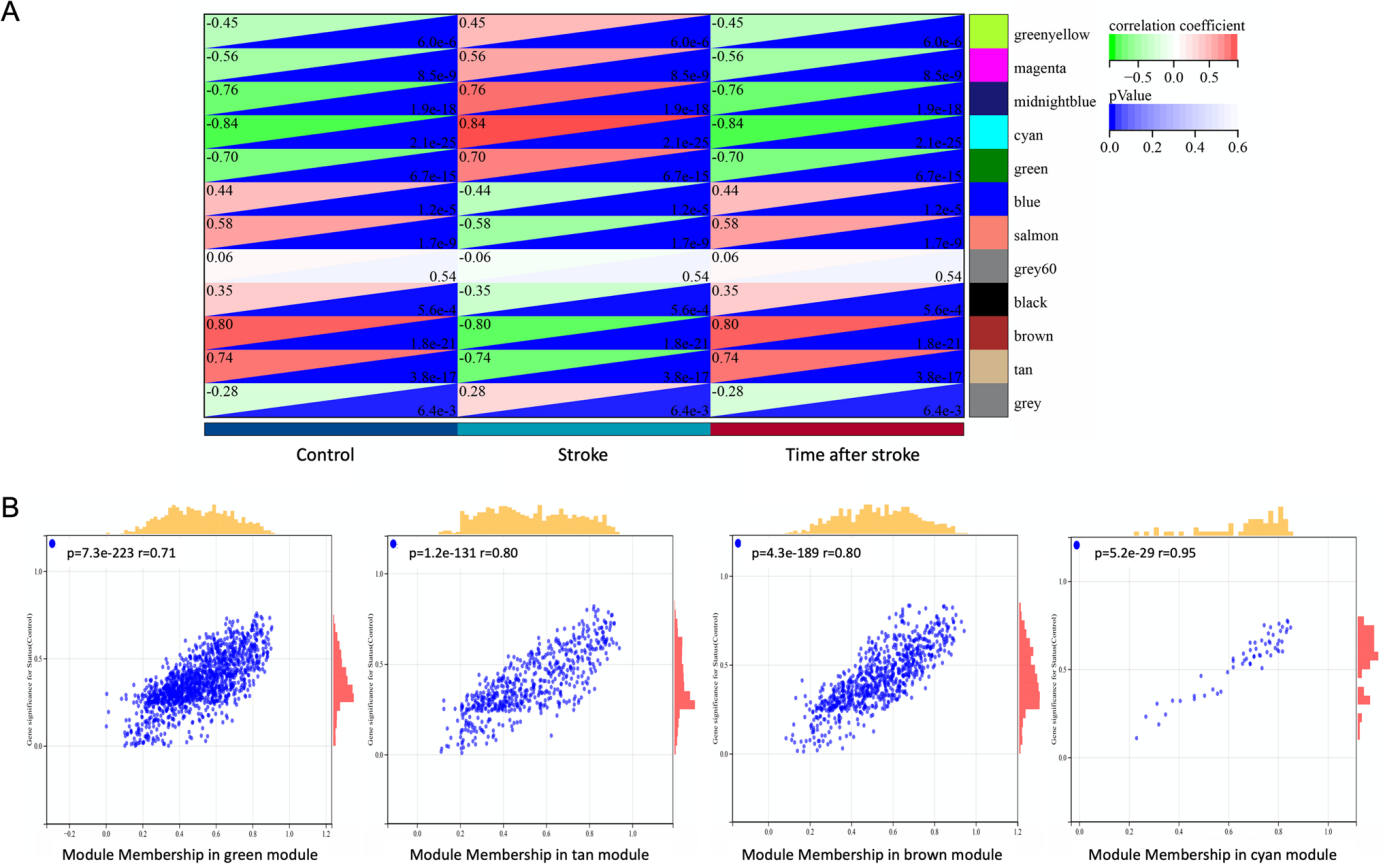
Identification of crucial modules associated with stroke. **A** Heatmap of the correlations between modules and stroke. Red represented a positive correlation, and green represented a negative correlation. The relevant *p* value and correlation coefficient are indicated in the figure, respectively. **B** Correlation plot between stroke and control group in module membership of green, tan, brown, and cyan modules

To identify the correlated module in cardioembolic stroke, the WGCNA analysis was applied after removing the duplicate values and filtering the genes by adj. *p* < 0.05 in GSE58924. 8535 genes were extracted and used *β* = 6 as the soft threshold power (Fig. 5A, B). The clustering dendrogram of the stroke and control is shown in Fig. 5C. Based on the weighted correlation, a hierarchical cluster analysis was performed to obtain 12 co-expression gene modules, represented by branches of the cluster tree and different colors (Fig. 5D, E). The 5 top modules with the largest absolute value of correlation coefficient, such as cyan (*r* = 0.84, *p* = 2.1e−25), brown (*r* = − 0.8, *p* = 1.8e−21), midnight blue (*r* = 0.76, *p* = 1.9e−18), tan (*r* = − 0.74, *p* = 3.8e−17), and green (*r* = 0.7, *p* = 6.7e−15), are shown in Fig. 6A. The correlations between module membership and gene significance in cyan, brown, tan, and green modules for stroke were calculated (Fig. 6B), and 241 hub genes were obtained and selected for further analysis.

### Identifying the SCFA Metabolism-Related Genes in Co-expression Modules of Stroke

**Fig. 7:**
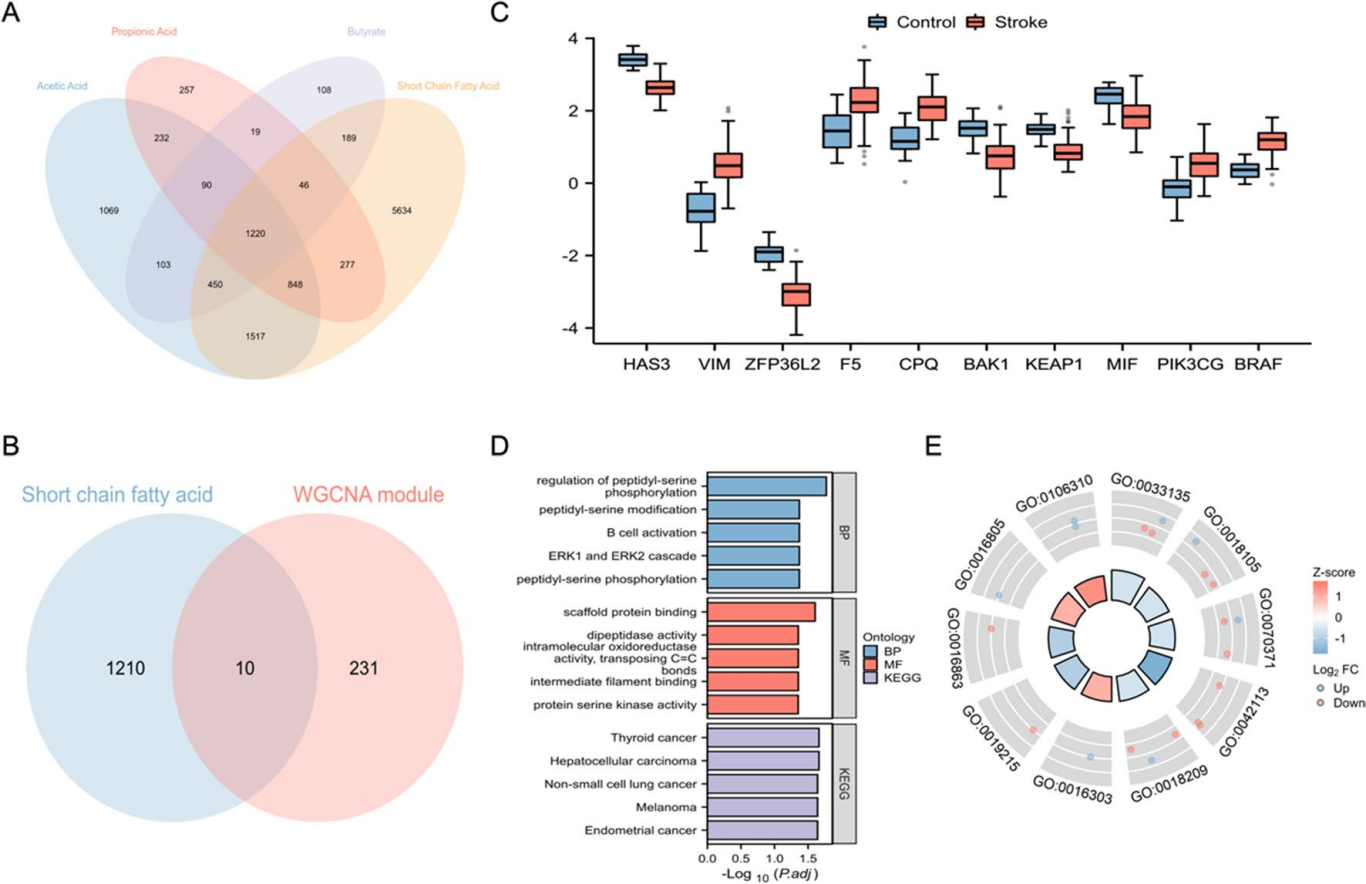
Identifying the SCFA-related hub genes in cardioembolic stroke. **A** The acetic acid, butyrate, propionic acid, and short-chain fatty acid genes searched in the GeneCards database were intersected in the Venn diagram. **B** The SCFA-related genes were intersected with the WGCNA co-expression module genes in the Venn diagram. **C** The expression of SCFA-related hub genes in cardioembolic stroke was shown in boxplots. **D** The GO and KEGG enrichment analysis of SCFA-related hub genes in cardioembolic stroke. **E** The GO and KEGG enrichment analysis combined with Log_2_FC values of SCFA-related hub genes in cardioembolic stroke was conducted in a circle diagram

To investigate the role of SCFA metabolism in developing and diagnosing stroke, first, the acetic acid (*n* = 5530), butyrate (*n* = 2226), propionic acid (*n* = 2990), and SCFA-related genes (*n* = 10182) were searched in the GeneCards database. The intersection of 4 gene sets is taken, and 1220 genes were identified (Fig. 7A). Then, 10 SCFA metabolism-related genes in co-expression modules of stroke were obtained by taking the intersections of 241 co-expression module genes of stroke and 1220 SCFA metabolism-related genes, including HAS3, VIM, ZFP36L2, CPQ, F5, PIK3CG, KEAP1, BAK1, MIF, and BRAF (Fig. 7B). Among these SCFA-related hub genes, HAS3, ZFP36L2, KEAP1, BAK1, and MIF were down-regulated, and VIM, CPQ, F5, PIK3CG, and BRAF were up-regulated compared to the control group (Fig. 7C). To better understand the function of these genes, we used enrichment analysis. GO analysis showed that these SCFA-related hub genes were related to the peptidyl-serine modification, ERK1 and ERK2 cascade, and B cell activation. The KEGG enrichment analysis suggested that the cancer-related pathways might be mainly involved in the biological pathways in stroke (Fig. 7D). Furthermore, these SCFA-related hub genes positively regulated the protein serine kinase activity, dipeptidase activity, and 1-phosphatidylinositol-3-kinase activity. In contrast, peptidyl-serine phosphorylation, ERK1 and ERK2 cascade, intermediate filament binding, and other enriched pathways were negatively regulated (Fig. 7E).

### Identifying the Diagnostic Markers of Stroke in SCFA-Related Hub Genes

**Fig. 8:**
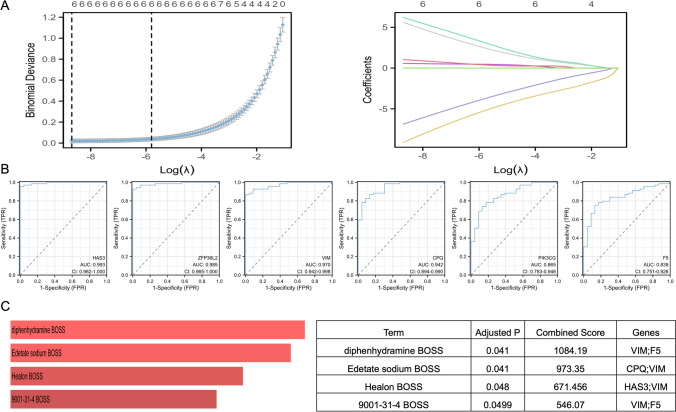
Identifying the diagnostic markers of stroke in SCFA-related hub genes. **A** Identification of SCFA-related hub genes via the LASSO model. LASSO coefficient profiles of 6 SCFA-related potential prognostic genes. Each curve corresponds to a gene. **B** ROC curves of 6 SCFA-related potential prognostic genes in cardioembolic stroke. **C** Identification of the candidate drugs targeting these 6 diagnostic markers of stroke with adj. *p* < 0.05

To investigate the role of SCFA metabolism in early stroke diagnosis, we performed SCFA-related hub genes screening through LASSO regression analysis. The results showed that 6 genes filtered with optimal lambda value were identified as diagnostic characteristic genes in stroke, namely, HAS3, VIM, ZFP36L2, CPQ, F5, and PIK3CG (Fig. 8A). To further test the diagnostic efficacy of the above genes, ROC curves were drawn to predict their diagnostic features. Figure 8B shows that the AUC values for all 6 genes were greater than 0.8, indicating that these 6 genes have excellent predictive capabilities. Among these, HAS3 (AUC = 0.993), ZFP36L2 (AUC = 0.985), VIM (AUC = 0.97), and CPQ (AUC = 0.942) had high diagnostic values (AUC > 0.9), while PIK3CG (AUC = 0.865) and F5 (AUC = 0.839) have a moderate accuracy (AUC = 0.7–0.9). These results indicated that these 6 genes could be promising markers for diagnosing stroke.

### Potential Drug Prediction

Finally, based on the drug and target information from the Enrichr platform, 4 candidate drugs targeting these 6 diagnostic markers of stroke with adj. *p* < 0.05 are shown in Fig. 8C. Diphenhydramine and 9001-31-4 target VIM and F5, edetate sodium targets CPQ and VIM, and healon targets HAS3 and VIM. Diphenhydramine is an antihistamine. It acts on the antihistamine H1 receptor, has a strong inhibitory effect on the central nervous system, and is widely used in treating allergic diseases of the skin and mucous membranes. Edetate sodium can inhibit the release of inflammatory mediators, thus achieving an anti-inflammatory effect. In the clinic, it is mainly used for the treatment of rheumatoid arthritis and ankylosing spondylitis. With this study, we have discovered that there may be new clinical applications for these drugs.

## Discussion

Stroke is a highly destructive and severe neurological condition with global implications. It can be classified into 2 specific types. Hemorrhagic strokes are caused by a rupture of a blood vessel inside the brain, while ischemic strokes arise from blockages of the arteries in the brain, both of which result in localized deprivation of oxygen and infliction of harm on brain tissue. Ischemic stroke, of the two, is the more prevalent. It is caused by interrupting cerebral blood flow due to thrombosis or embolism. The mechanisms of ischemic stroke are complex and include effects on cellular excitotoxicity, mitochondrial dysfunction, oxidative stress, neuroinflammation, and cell death. To date, tissue plasminogen activator (tPA), a thrombolytic drug, is the only FDA-approved medicine for ischemic stroke. Prophylactic therapies comprise anticoagulants, blood pressure-reducing, and cholesterol-lowering drugs. The treatments available for ischemic stroke had to be treated with specialized devices within 10 h of stroke onset. In addition, rtPA and clot repair only restore blood flow and do not target cellular damage mechanisms or promote healing. It is crucial to conduct further research to establish the most effective treatment approach. Additionally, approximately one out of four stroke survivors suffer a second stroke within 5 years [28]. Therefore, researching the management of stroke rehabilitation pathways can assist in reducing the impact of disability caused by stroke.

Immune dysfunction following a stroke is a well-established phenomenon encompassing immune activation and immunosuppression. The activation of the immune system after a stroke may be attributed to the disruption of the blood-brain barrier, which permits contact between CNS antigens that are normally kept separate immunologically, and the systemic immune system, resulting in an autoimmune response. In contrast, post-stroke immunosuppression could be linked to an immune response that triggers the hypothalamic-pituitary-adrenal axis and the sympathetic nervous system to carry out an immune-suppressive function. Promoting both inflammatory and anti-inflammatory immune responses is necessary to achieve a dynamic balance in the internal environment. A disturbance in this system may result in secondary tissue damage. The influence of various inflammatory factors on the brain during and after ischemia is not fully comprehended. Investigating biomarkers and therapies related to immunomodulation can offer potential optimism for detecting and managing this ailment [29].

To the best of our knowledge, the evaluation of SCFA metabolism and stroke development through bioinformatics methods has not been reported in previous articles. Here, we evaluated the pathogenesis of stroke using GSEA and immune cell infiltration. The relative percent of immune cells revealed that patients suffering cardioembolic stroke exhibit greater proportions of neutrophils and monocytes alongside lower proportions of NK cells, mast cells, and M1-type macrophages (Fig. 2A). The cardioembolic stroke patients have a higher correlation to these 22 kinds of immune cell infiltration compared to the control group. Research has demonstrated that following a stroke, brain tissue triggers an inflammatory response. This leads to the activation of neutrophils, which migrate to the ischemic area and initiate innate immunity. Unfortunately, this defense overload can also cause damage to the brain tissue and blood vessels. This suggests that patients with cardiogenic stroke may recruit peripheral immune cells to infiltrate the center and further aggravate the inflammatory response. Our analysis has reached the same conclusion. GSEA analysis showed that stroke pathogenesis is mainly associated with neutrophil degranulation and B cell regulation (Fig. 3A, B). These are consistent with the current mechanism of stroke pathogenesis, which is that a novel B cell phenotype, known as the macrophage-like B cell, has been identified in the brain. This type of B cell has enhanced phagocytosis and chemotaxis abilities. It can contact, phagocytose, and internalize post-ischemic myelin fragments via TREM2. Additionally, it can recruit peripheral immune cells by releasing multiple chemokines via the CCL pathway [30]. Furthermore, our examination of DEGs indicated the indispensability of these genes in lymphocyte differentiation, B cell differentiation, and the regulation of immune response signaling pathways. For instance, a microtubule-associated motor protein, kinesin-like family member 20A (KIF20A), is highly expressed in immature hematopoietic cells and functions as a prognostic factor of the tumor by correlating with Th2 and Treg cells [31, 32]. CDKN3, TPX2, and HMMR are also linked to immune cell infiltration in several types of tumors [33–35].

Over the past decade, evidence has highlighted the impact of the human gut microbiota and its metabolites on health and disease. The stroke and other neurological diseases are thought to be associated with microbiota disruption. Several studies have demonstrated that dysbiosis of gut bacteria can cause pathological changes and alter the formation of bacterial metabolites, leading to the immune system and metabolic dysregulation and the development of clinical risk factors for stroke (e.g., Western-style high-fat diet, hypertension, T2DM, obesity, insulin resistance, metabolic syndrome, dyslipidemia, atherosclerosis, aging, vascular dysfunction, inflammation, leaky gut, and alcohol excess) [36, 37]. SCFAs such as butyric, acetic, and succinic acids are the main metabolites produced by bacterial fermentation of dietary fiber within the gastrointestinal tract. They are potential immunomodulatory factors that interact with various immune cells, mediate systemic inflammatory responses, and are intimately involved in the passage of nutrients from the circulation to the brain. They also influence the structural and functional integrity of microglia to be associated with neuroinflammation, promote neuronal growth and synaptic plasticity, and play an essential role in brain development. For example, SCFAs facilitate the mitosis of neuronal cells, increase growth rate, and regulate early neurological outcomes [38].

SCFAs have also been shown to have a significant protective effect against neurodegenerative diseases. They effectively alleviate ischemic stroke by transplantation of SCFA-rich cecal fecal bacteria and concomitant supplementation with butyric acid [39]. Moreover, in vivo and in vitro studies have shown that acetate modulates the expression levels of inflammatory factors and related signaling pathways in astrocytes and attenuates the activation of glial cells in a rat model of neuroinflammation [40]. Similarly, butyrate induces morphological and functional changes in microglia to homeostasis and inhibits lipopolysaccharide-induced expression of pro-inflammatory factors as well as depressive and manic symptoms [41, 42]. And butyrate inhibits inflammation in rat microglia, hippocampal tissue, and astrocytes [43]. In addition, butyrate can promote neuronal plasticity, enhance memory, restore cognitive impairment, reduce neurotoxicity, neuroinflammation, and behavioral abnormalities, and alleviate various CNS disease symptoms by inhibiting histone deacetylase activity [44, 45]. In summary, SCFA may affect brain function in test animals by modulating neurodevelopment and microglia activation.

In recent years, the pathogenic association between stroke-induced gut dysbiosis and poor treatment outcomes has been actively explored in the hope of finding more effective treatments. Our study used a series of integrated bioinformatics analyses and machine learning algorithms to evaluate the diagnostic value for SCFA-related hub genes in stroke patients. After taking the intersection of SCFA metabolism-related genes and the co-expression WGCNA modules of stroke, 10 SCFA-related hub genes were obtained, and 6 of them showed high diagnostic efficacy through LASSO regression and ROC analysis, including HAS3, VIM, ZFP36L2, CPQ, F5, and PIK3CG. Of these, VIM (vimentin) is a cytoskeletal intermediate filament protein positively linked to atherosclerosis and thrombosis [46, 47]. Recently published literature has shown that vimentin can act as a receptor for many bacteria and viruses and play a role in brain-pathogen interactions. It is involved in recognizing gastrointestinal *Escherichia coli* and mediating the activation of innate immune signaling [48] and is correlated to the genus Oscillospora, which is associated with health in humans and mice [49]. CPQ (Carboxypeptidase Q), a carboxypeptidase enzyme, has been found to have protein homodimerization activity and carboxypeptidase activity. The relation between CPQ and stroke remains unexplored. However, the carboxypeptidase U has been reported to elevate the risk of microvascular thrombosis [50]. It suggests that CPQ might be a promising therapeutic target for stroke. The PIK3CG gene encodes the gamma chain of the PI3/PI4-kinase. PI3K/AKT signaling has been reported to play a central role in blood-brain barrier dysfunction in stroke [51]. It also correlates with inflammatory responses, oxidative stress, apoptosis, cellular autophagy, and vascular endothelial function in ischemic stroke [52]. In addition, extensive research has shown the contribution of PI3K/Akt/mTOR signaling cascades in gastrointestinal disorders [53]. Although these targets have been reported to be associated with stroke and gut flora, no study has investigated the effect of targeting gut flora to treat stroke. Based on the drug and target information from the Enrichr platform, 4 candidate drugs targeting these 6 diagnostic markers of stroke are diphenhydramine, 9001-31-4, edetate sodium, and healon, which were previously used for blood clotting and inflammatory response. Our study discovered promising new clinical applications for these existing drugs that could be repurposed to treat stroke.

In conclusion, we have identified several hub genes related to stroke that have the potential to aid in the diagnosis and comprehension of its development. Therefore, these hub genes should be further investigated in future stroke research.

## Data Availability

No datasets were generated or analyzed during the current study.
